# ﻿New subgenera and a new species of the genus *Raphignathus* Dugès (Prostigmata, Raphignathidae), with taxonomic notes on the genus *Neoraphignathus* Smiley & Moser

**DOI:** 10.3897/zookeys.1176.106224

**Published:** 2023-08-23

**Authors:** Eid Muhammad Khan, Muhammad Kamran, Jawwad Hassan Mirza, Fahad Jaber Alatawi

**Affiliations:** 1 Acarology Research Laboratory, Department of Plant Protection, College of Food and Agriculture Sciences, King Saud University, Riyadh, Saudi Arabia King Saud University Riyadh Saudi Arabia

**Keywords:** Acari, new combinations, predatory mite, Raphignathoidea, Saudi Arabia

## Abstract

Four new subgenera in the genus *Raphignathus* Dugès are hereby proposed: Raphignathus (Raphignathus), **subgen. nov.**, Raphignathus (Monoraphignathus), **subgen. nov.**, Raphignathus (Diraphignathus), **subgen. nov.**, and Raphignathus (Triraphignathus), **subgen. nov.** These subgenera are diagnosed by the number of setae on the interscutal membrane of females. A new species, R. (D.) neohecmatanaensis**sp. nov.**, is described and illustrated based on females collected from *Ziziphusspina-christi* Mill. (Rhamnaceae). The taxonomic status of the monotypic genus *Neoraphignathus* Smiley & Moser and three species (*R.evidus*, *R.hsiufui*, and *R.johnstoni*) are discussed. A key to world species of the family Raphignathidae is given.

## ﻿Introduction

Members of the family Raphignathidae Kramer (Prostigmata, Raphignathoidea) are active predators feeding on small arthropods (Meyer and Ueckermann 1989). They are mostly found in humus soil under dense bushes, leaf litter, lichens, and mosses and on a wide range of plants (Fan and Zhang 2005). Raphignathids have cervical peritremes and contiguous coxae and are divided into two genera: *Raphignathus* Dugès (76 species) and *Neoraphignathus* Smiley & Moser (one species, *N.howei* Smiley & Moser) (Smiley and Moser 1968; Khanjani et al. 2013). These genera are mainly differentiated by the presence and absence of dorsal shields, respectively. *Raphignathus* species have a worldwide distribution, whereas *Neoraphignathus* is only known from Louisiana, USA (Beron 2020).

The genus *Raphignathus* (type species: *R.ruberrimus* Dugés) was diagnosed as having three, or sometimes four, dorsal shields (Atyeo et al. 1961). While discussing the chaetotaxy of the superfamily Raphignathoidea, Atyeo et al. (1963) provided a general description of the genus *Raphignathus*. They considered the number of setae on prodorsal shields and on interscutal membrane as important taxonomic characteristics. In addition, the number of setae on the interscutal membrane was also used to differentiate among the species in recently published diagnostic keys (Nasrollahi et al. 2018; Pishehvar and Khanjani 2021). The validity of some *Raphignathus* species has been questionable due to ontogenetic development, and more females need to be collected and observed (Dönel and Doğan 2011). *Raphignathusbroomicus* Podder was considered a species inquirenda due to uncertain and doubtful characters (Doğan and Erman 2019). Until now, most of published work on the family Raphignathidae has been regional and includes identification keys for China, India, Iran, and Turkey (Fan and Yin 2000; Doğan and Erman 2019; Pishehvar and Khanjani 2021).

In the present study, four new subgenera of *Raphignathus* are erected based on the prominent and consistent morphological character (number of setae on interscutal membrane). A new species, R. (D.) neohecmatanaensis sp. nov. is described and illustrated based on females. Some taxonomic notes on the monotypic genus, *Neoraphignathus* Smiley & Moser, and the identity of three species (*R.evidus*, *R.hsiufui*, and *R.johnstoni*) are concisely discussed. A diagnostic key to the world species is also provided.

## ﻿Material and method

All published taxonomic literature on the family Raphignathidae was critically reviewed to confirm the validity of the species, subgeneric divisions and to prepare a diagnostic key of world species. The new raphignathoid species was collected by shaking foliage of *Ziziphusspina-christi* Mill. (Rhamnaceae) over a white sheet of paper; mite specimens were preserved in small vials containing 70% ethanol. The specimens were permanently mounted on glass slides in Hoyer’s medium and identified under a phase-contrast microscope (BX51, Olympus, Tokyo, Japan). All measurements of the holotype specimen are given in micrometers (μm), followed by those of paratypes in the parenthesis. The terminology and abbreviations used in the description of the new species follow those of Kethley (1990) and Grandjean (1939, 1944, 1946). The holotypes and paratypes were deposited at the King Saud Museum of Arthropods (**KSMA**, Acarology section), Department of Plant Protection, College of Food and Agriculture Sciences, King Saud University, Riyadh, Saudi Arabia.

## ﻿Results

Four new subgenera of the genus *Raphignathus* are proposed: Raphignathus (Raphignathus), subgen. nov., Raphignathus (Monoraphignathus), subgen. nov., Raphignathus (Diraphignathus), subgen. nov., and Raphignathus (Triraphignathus), subgen. nov., on the basis of the number of setae on the interscutal membrane, a prominent and consistent morphological character. A new species, R. (D.) neohecmatanaensis sp. nov., is described and illustrated based on adult females. Furthermore, taxonomic notes on the status of the monotypic genus, *Neoraphignathus* Smiley & Moser, and the identity of three species (*R.evidus*, *R.hsiufui* and *R.johnstoni*) are discussed. A diagnostic key to the world species is also presented.

### ﻿Family Raphignathidae

Taxon classificationAnimaliaProstigmataRaphignathidae

Kramer, 1877

1464681B-3E4D-5CAF-92DB-EB3727AA4A1C

Raphignathidae Kramer, 1877: 215

#### Type genus.

*Raphignathus* Dugès, 1834: 53

#### Diagnosis

(based on Krantz and Walter (2009) and Fan and Zhang (2005)). Peritremes linear, not imbedded in dorsal surface of stylophore; paired peritremes running laterally from base of stylophore to make short loops in collar membrane between gnathosoma and podosoma; coxae II and III contiguous; stigmata opening at base of chelicerae.

##### ﻿Taxonomic divisions of the genus *Raphignathus*

The presence or absence of prodorsal shields are diagnostic for the differentiation of the two existing raphignathid genera. Atyeo (1963) discussed in detail the chaetotaxy of the superfamily Raphignathoidea while describing seven *Raphignathus* species. This author also provided comprehensive diagnosis of *Raphignathus* and stated that the number of setae on the shields and the interscutal membrane is a consistent and important diagnostic character. The number of setae on the interscutal membrane is considered to be a strong character and has been used in identification keys to distinguish species (Nasrollahi et al. 2018; Pishehvar and Khanjani 2021). Through our extensive study of the literature of all 76 *Raphignathus* species, we find that the number of setae on the interscutal membrane can be used to erect subgenera.

In the current study, we categorize species of the genus *Raphignathus* into four new subgenera based on the number of setae on the interscutal membrane. These four subgenera are Raphignathus (Raphignathus), subgen. nov. without setae (11 spp.), Raphignathus (Monoraphignathus), subgen. nov. with one seta (14 spp.), Raphignathus (Diraphignathus), subgen. nov. with two setae (33 spp.), and Raphignathus (Triraphignathus), subgen. nov. with three setae (10 spp.).

### ﻿ Raphignathus (Raphignathus)

Taxon classificationAnimaliaProstigmataRaphignathidae

9647ECD3-9FB0-5F1B-8310-4C401721C64D

https://zoobank.org/4A81EA35-DC89-4971-BD24-0459A27A8621

#### Type species.

*Raphignathusruberrimus* Dugès, 1834: 53.

#### Diagnosis.

Interscutal membrane without setae.

#### Etymology.

The subgeneric epithet refers to the nominotypical subgenus.

### ﻿ Raphignathus (Monoraphignathus)

Taxon classificationAnimaliaProstigmataRaphignathidae

E0F96820-6E72-5ADB-A958-0652A1A3C16A

https://zoobank.org/D332A8E8-0390-4D39-95D4-A8C6E7E0B8D5

#### Type species.

*Raphignathusbathursti* Meyer & Ryke, 1960: 229.

#### Diagnosis.

Interscutal membrane with one pair of setae.

#### Etymology.

The subgeneric epithet refers to the one pair of setae on interscutal membrane.

### ﻿ Raphignathus (Diraphignathus)

Taxon classificationAnimaliaProstigmataRaphignathidae

9FADAE20-20A8-59F6-9FE7-7314FC6A9584

https://zoobank.org/0D7C8B12-10E8-4019-A2FA-591934FD9B17

#### Type species.

*Raphignathusgracilis* (Rack, 1962): 281.

#### Diagnosis.

Interscutal membrane with two pairs of setae.

#### Etymology.

The sub-generic epithet refers to the two pairs of setae on interscutal membrane.

The species included in this new subgenus are widely distributed over the world.

### ﻿ Raphignathus (Triraphignathus)

Taxon classificationAnimaliaProstigmataRaphignathidae

56BD0366-5B72-5D0D-8593-9BE41561D9EE

https://zoobank.org/80C632B9-6521-47BB-A6E2-3EA44EE761DD

#### Type species.

*Raphignathusdomesticus* Shiba, 1969: 157.

#### Diagnosis.

Interscutal membrane with three pairs of setae.

#### Etymology.

The subgeneric epithet refers to the interscutal membrane with three pairs of setae.

##### ﻿Notes on the taxonomic status of the genus *Neoraphignathus*

To date, the family Raphignathidae has included two genera, *Raphignathus* and *Neoraphignathus*, which have been differentiated based on the presence or absence of shields on the dorsum. The monotypic genus, *Neoraphignathus* (type species: *N.howei* Smiley & Moser) was erected in 1968, based on a single female holotype specimen without detailed description and illustration. Based on observations and the collection of the immature specimens of the genus *Raphignathus*, prodorsal shields are weakly developed or absent in immatures. Atyeo et al. (1961) has reported that dorsal shields are sometime feebly developed. Since its first description, the type species, *N.howei*, has not been redescribed, nor have new *Neoraphignathus* species been described. We suggest that the type specimen of *N.howei* be re-examined and also that more specimens be collected from the type locality to confirm the absence of a dorsal shield to confirm the validity of *Neoraphignathus*.

##### ﻿Notes on the validity of *Raphignathusevidus, R.hsiufui*, and *R.johnstoni*

The taxonomic identity of *Raphignathusevidus* Fan, *R.hsiufui* Fan, and *R.johnstoni* Womersley are doubtful. These species were originally described based on single specimens, minor differential characteristics (i.e. number of dorsal setae on the lateral prodorsal shield; all three species have two pairs of setae on lateral shields), and small opisthosomal shields. In contrast, all other *Raphignathus* species have three pairs of setae on the lateral prodorsal shields along with the pores (*ia*). The immature stages of *Raphignathus* gradually develop the prodorsal shields, striation patterns, and leg setae (Fan and Yin 2000). For instance, the immature stages of *R.giselae*, *R.lenis*, and *R.caspicus* each have two setae on the lateral prodorsal shields (three setae in adult) and small lateral prodorsal shields with weakly developed striations. Moreover, we also observed the immatures from more than 10 populations of *Raphignathus* and found reduced size of weakly sclerotized lateral prodorsal shields and setae set on the edges of shields. Based on this evidence, *R.evidus*, *R.hsiufui*, and *R.johnstoni* should be revised and more specimens collected to confirm their validity.

##### ﻿New species

### ﻿ Raphignathus (Diraphignathus) neohecmatanaensis

Taxon classificationAnimaliaProstigmataRaphignathidae

sp. nov.

4E0A4286-D871-5265-AAD6-99D360ED0DEF

https://zoobank.org/12E13CEE-478F-4A3F-A47E-B3C7F34C73FA

Figs 1 , 2 , 3 , 4 , 5–8

#### Diagnosis.

**Female**: endopodal shield absent between coxae I–IV; two small shields present posterolateral to median prodorsal shield; palp femora with two setae; femora 6–5–3–2; genua 5(+κ)-5(+κ)–4–4; tibiae 5(+φρ)–5(+φρ)–4(+φρ)–4 (+φρ); tarsi 21(1ω) –20(1ω)–15–14.

#### Description.

**Female** (*n* = 4). Idiosoma oval, length of body (including gnathosoma) 533 (525–545); width of body 345 (338–353).

**Figure 1.**
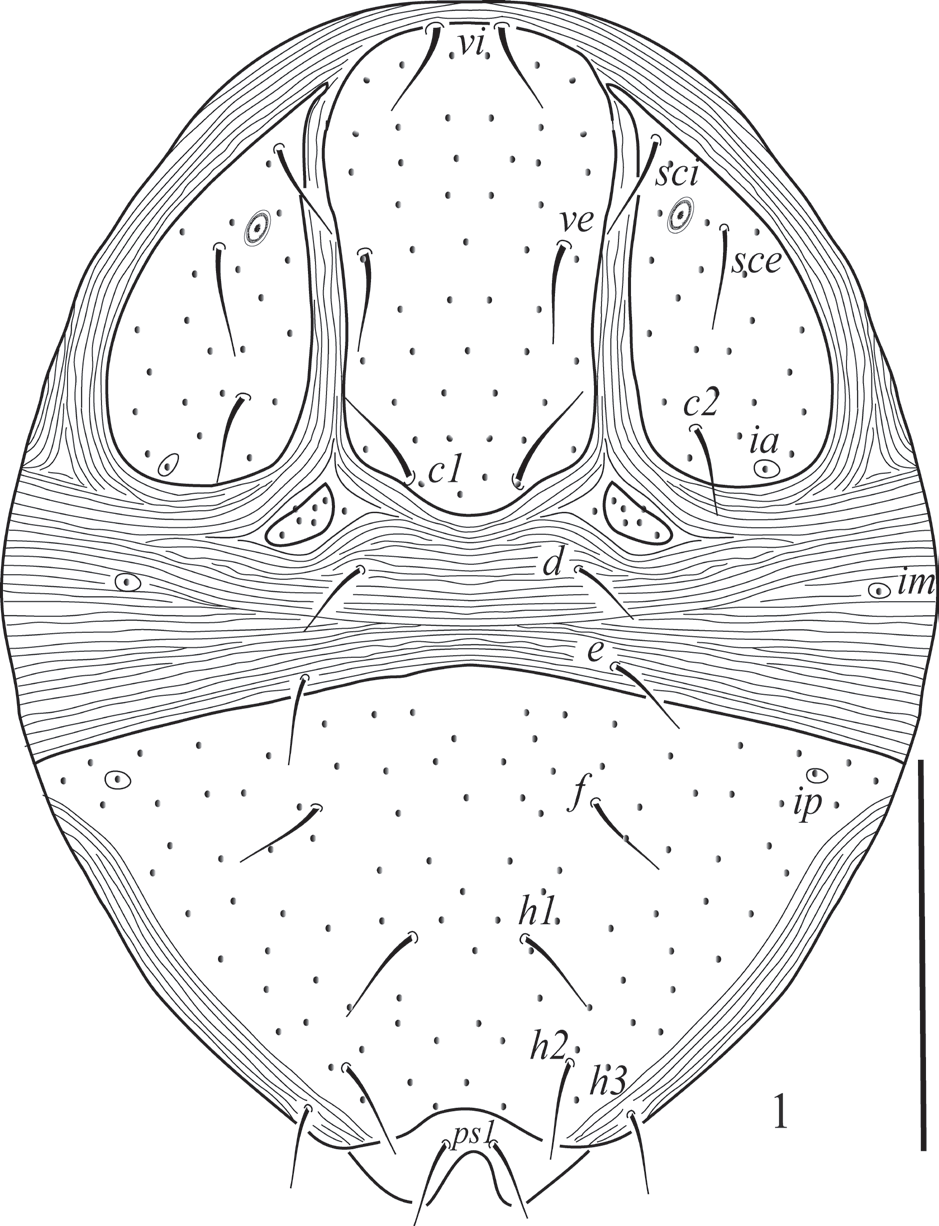
Raphignathus (Diraphignathus) neohecmatanaensis sp. nov. (female), dorsum. Scale bar: 100 µm.

***Dorsum*** (Fig. 1). Propodosoma with one medial and two lateral shields each containing three setae; medial sclerite with setae *vi*, *si* and *c1*; paired ovoid lateral shields each with an eye, one cupule (*ia*) and *sci*, *sce* and seta *c2* seta; opisthosomal setal pairs *d1*, *e1* and the pair of cupuli (*im*); posterior opisthosomal shield large, rectangular, bearing four pairs of setae (*f1*, *h1–3*) and one pair of the cupule (*ip*); all dorsal shields finely punctate; dorsal body setae setiform, smooth and acute; pseudanal setae *ps1* dorsally located. Lengths of dorsal setae: *vi* 25 (23–27), *ve* 28 (26–30), *sci* 27 (26–29), *sce* 28 (26–29), *c1* 23 (17–19), *c2* 25 (19–23), *d1* 21 (20–23), *e1* 22 (21–23), *f1* 21 (19–22), *h1* 24 (23–25), *h2* 25 (23–25), *h3* 22 (21–24), *ps1* 23 (21–25); distances between dorsal 195 setae: *vi–vi* 27 (29–31), *sci–sci* 123 (118–128), *vi– sci* 70 (66–72), *sce–c2* 68 (65–72), *c1–c1* 30 (28–32), *d1–d1* 99 (92–101), *c1–d1* 53 (49–55), *d1–e1* 22 (21–24), *f1–f1* 88 (83–90), *e1–f1* 65 (61–68), *h1–h1* 45 (42–48), *h1–h2* 43 (41–46), *h2–h2* 77 (72–80), *h3–h3* 101 (98–106).

**Figure 2.**
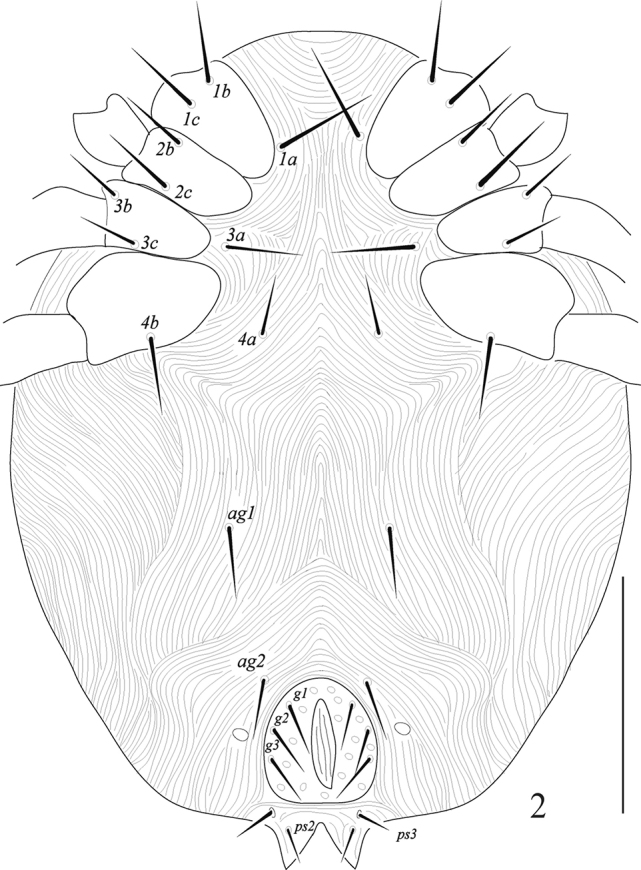
Raphignathus (Diraphignathus) neohecmatanaensis sp. nov. (female), venter. Scale bar: 100 µm.

***Venter*** (Fig. 2). Venter entirely striated, without punctations; coxisternal shields absent (Fig. 2); ventral setae lengths: *1a* 42 (41–44), *1b* 38 (35–39), *1c* 34 (33–36), *2b* 36 (30–34), *2c* 32 (30–34), *3a* 24 (23–26), *3b* 18 (17–20), *3c* 36 (34–38), *4a* 22 (23–27), *4c* 38 (35–40); two pairs of aggenital setae (*ag1–2*) with one cupule (*ih*) on each side of the genital shield; anal opening and genital shields separate; genital shield prominent with a few punctations, bearing three pairs of genital setae (*g1–3*); anal opening terminal, with three pairs of setae (*ps1*–3), *ps1* dorsal *ps3* and *ps2* ventral; ventral setal lengths: *ag1* 29 (27–32); *ag2* 27 (25–28); *g1* 31 (28–33); *g2* 25 (23–28); *g3* 20 (19–22); *ps2* 22 (21–23); *ps3* 21 (20–22). Distances between ventral setae: *1a–1a* 55 (53–58), *3a–3a* 115 (97–109), *4a–4a* 70 (68–73), *ag2–ag2* 42 (41–43), *g1–g1* 38 (35–40), *g2–g2* 52 (48–55), *g3–g3* 75 (74–75), *2b–2c* 30 (25–29), *1a–3a* 50 (49–53), *3a–4a* 72 (68–75), *4a–ag1* 85 (82–88), *ag1–ag2* 65 (63–68), *ag2–g1* 63 (60–65), *g1–g2* 15 (14–17), *g2–g3* 22 (21–25), *ag–g1* 42 (40–45), *g3–ps3* 16 (15–18), *ps2– ps3* 16 (14–18).

**Figure 3.**
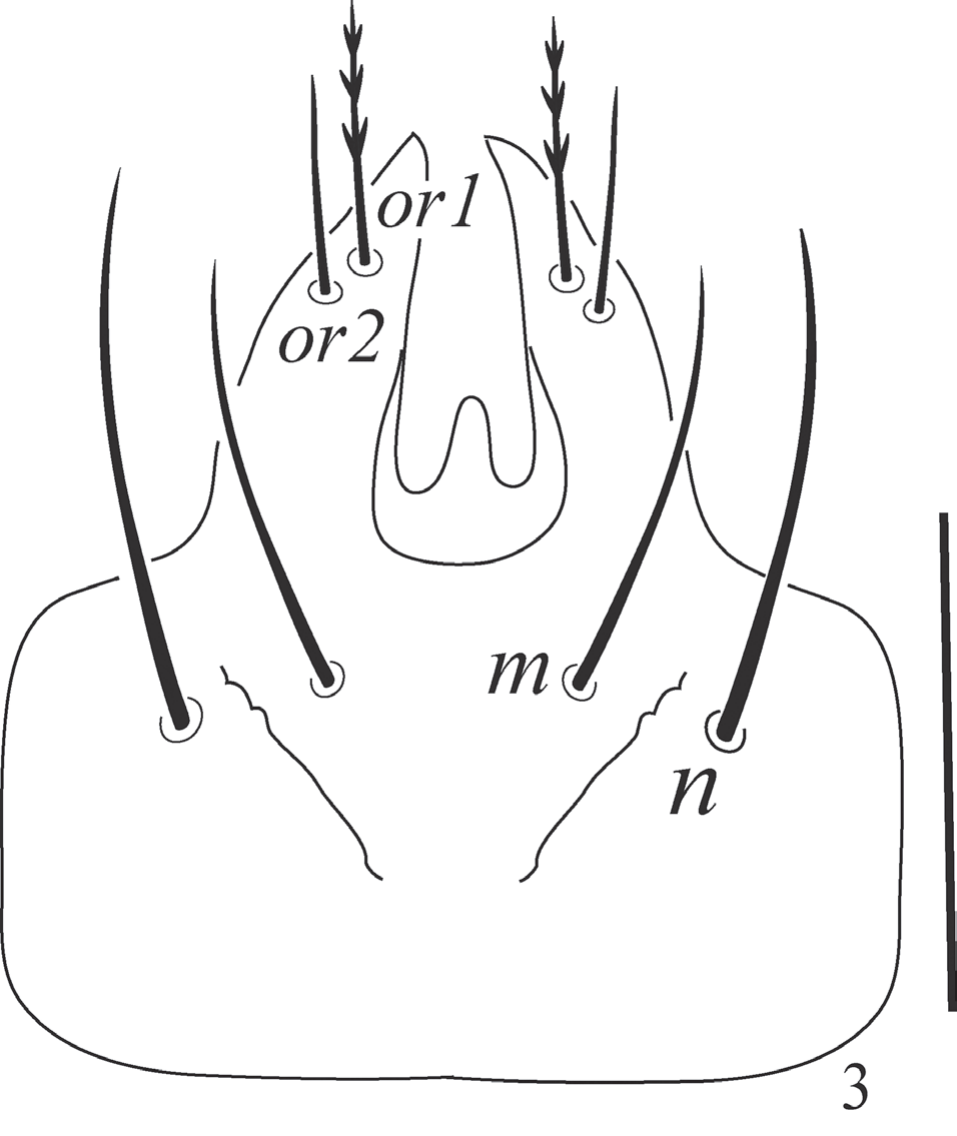
Raphignathus (Diraphignathus) neohecmatanaensis sp. nov. (female), gnathosoma. Scale bar: 50 µm.

**Figure 4.**
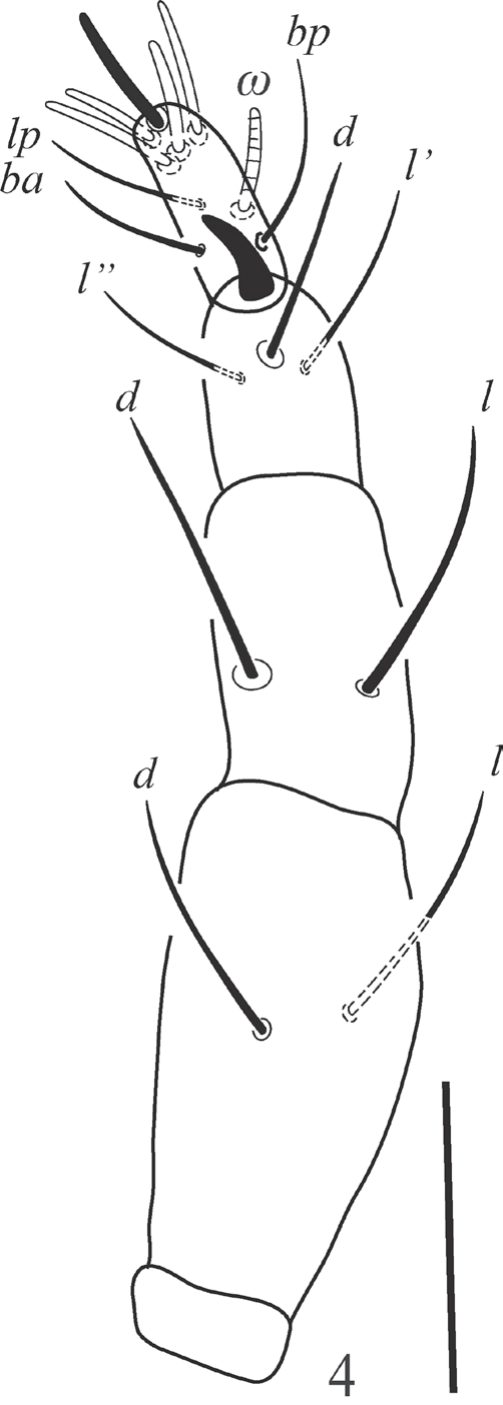
Raphignathus (Diraphignathus) neohecmatanaensis sp. nov. (female), palp. Scale bar: 20 µm.

***Gnathosoma*** (Figs 3, 4). Ventral infracapitular with two pairs of very long setae (*m* and *n*), *m* 40 (39–42), *n* 52 (49–54) and two pairs of pilose adoral setae (*or1–2*), *or1* 23 (21–24), *or2* 20 (19–22) (Fig. 3); stylophore conical and striated; palp chaetotaxy (femur-tarsus) as follows: 3–2–4+1 claw 4+1ω+4 eupathidia (ζ) (Fig. 4).

**Figures 5–8.**
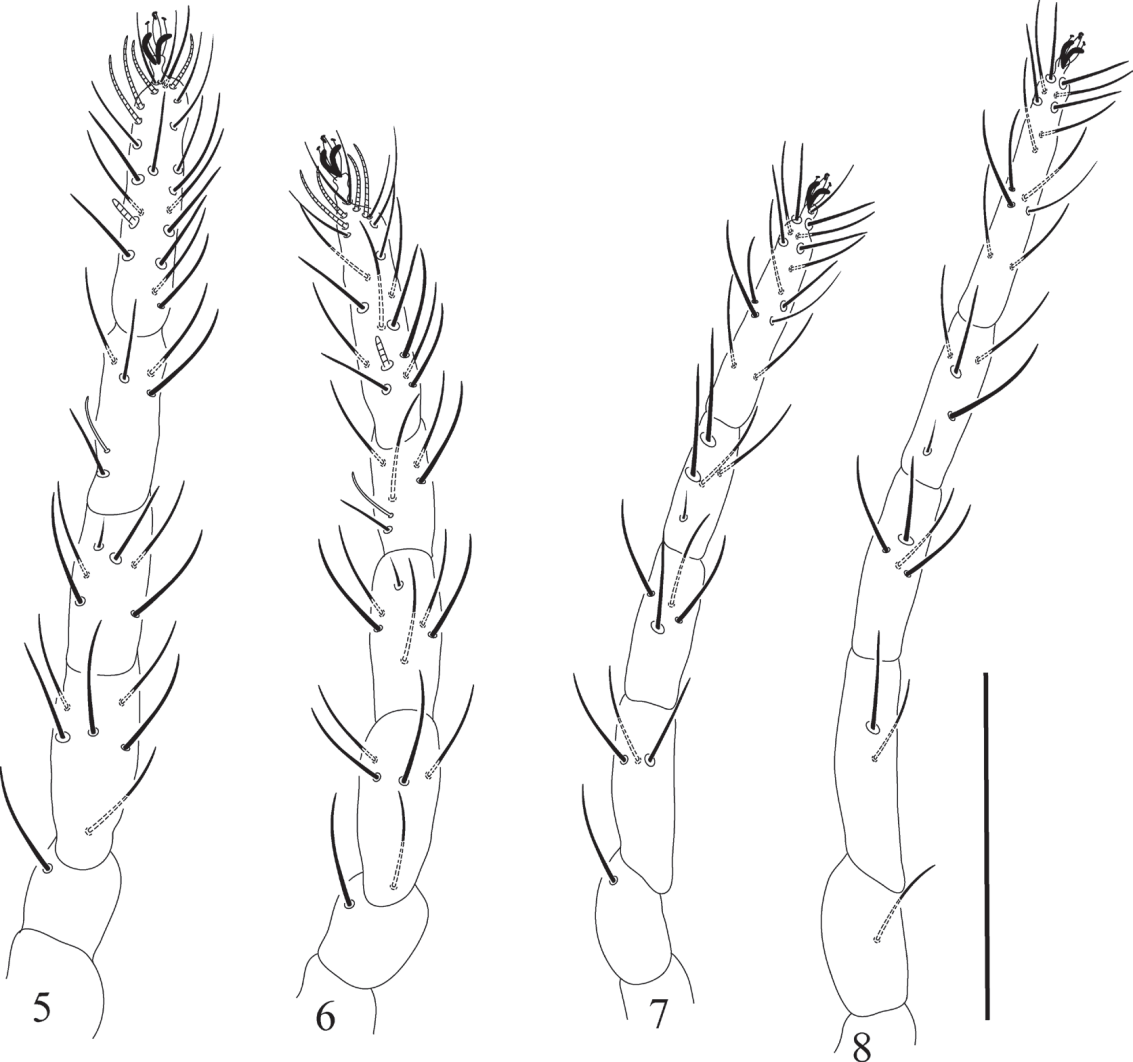
Raphignathus (Diraphignathus) neohecmatanaensis sp. nov. (female), Legs I–IV. Scale bar: 20 µm.

***Legs*** (Figs 5–8). Length of legs I–IV (without coxae): 340 (328–348); 270 (276–286); 325 (317–328); 375 (367–384), respectively. Chaetotaxy on legs I–IV (solenidia in parentheses and not included in setal counts): coxa 2–2–2–1; trochanter 1–1–2–1; femora 6–5–3–2; genua 5(+κ)-5(+κ)–4–4; tibiae 5(+φρ)–5(+φρ)–4(+φρ)–4(+φρ); tarsi 21(1ω) –20(1ω)–15–14.

#### Male and immature stages.

Unknown.

#### Type materials.

***Holotype*** female and three paratype females, Faifa, Jazan, 24°30.412'N, 39°36.578'E, 8 Oct., 2020, collected from *Ziziphusspina-christi* Mill. (Rhamnaceae) by Eid M. Khan, Jawwad H. Mirza & Hafiz S. Mushtaq.

#### Etymology.

The specific epithet is in reference to the similarity of the new species to R. (D.) hecmatanaensis; *neo* = new.

#### Remarks.

Raphignathus (D.) neohecmatanaensis sp. nov., belongs to the subgenus Diraphignathus subgen. nov. The new species resembles to R. (D.) hecmatanaensis Khanjani & Ueckermann in having two pairs of setae (*d1* and *e1*) on the interscutal membrane, two setae on the palp femora, and two small plates present posterolateral to the median prodorsal shield. However, the new species differs from R. (D.) hecmatanaensis in the absence of an endopodal shield (vs present), femur IV with two setae (vs three), and leg tarsus I with one solenidion (vs two solenidia).

### ﻿Key to genera, subgenera, and all known species of the family Raphignathidae

Five species are not included in the key. *Raphignathuslongimanus* (Koch), *R.impressus* (Koch), *R.hispidus* (Dugès), and *R.deserticula* (Trägårdh) because their descriptions are incomplete, and *R.lanuginosus* Atyeo is excluded, as it was described on the male.

**Table:** 

1	Dorsum with well-developed shields, one medial, one pair of lateral shields on prodorsum and one hysterosomal shield	**Genus *Raphignathus* [2**]
–	Dorsum without shields	**Genus *Neoraphignathus* , *N.howei* Smiley & Moser**
2	Interscutal membrane without setae, hysterosomal shield with six pairs of setae	**Raphignathus (Raphignathus) subgen. nov. [3**]
–	Interscutal membrane with ≥1 setae	**13**
3	Opisthosoma or opisthosomal shield reticulated	**4**
–	Opisthosomal shield smooth or otherwise, not reticulated	**6**
4	Prodorsal shields reticulated	**R. (R.) crustus Fan & Zhang, New Zealand**
–	Prodorsal shields smooth or punctate	**5**
5	Tibia I with 5 +2φ, Tibia III with 5+1φ, dorsal body setae comparatively long (54–74)	**R. (R.) kurdistaniensis Nasrollahi, Khanjani & Mirfakhraei, Iran**
–	Tibia I with 5 +1φ, Tibia III with 4+1φ, dorsal body setae comparatively short (24–36)	**R. (R.) darjeelingensis Gupta, India**
6	Opisthosoma without distinct shield; setae *d1*, *e1*, and *f1* very minute, 1/3–1/2 length of setae *v1*	**R. (R.) guajavae Gupta, India**
–	Opisthosoma with distinct opisthosomal shield; setae *d1*, *e1*, and *f1* at least 2/3 length of setae *v1*	**7**
7	Genu I and II each with a large leaf-like solenidion	**R. (R.) pycnonotus (Gupta & Paul), India**
–	Genu I and II each with a small, slender solenidion	**8**
8	Setae *c1* short, reaching 1/3 length of interscutal membrane, far behind the anterior margin of opisthosomal shield; tarsus IV with large solenidion	**R. (R.) inornata (Gupta & Paul), India**
–	Setae *c1* crossing interscutal membrane and anterior margin of opisthosomal shield; tarsus IV with small solenidion	**9**
9	Tibia I with one solenidion	**10**
–	Tibia I with two solenidia	**12**
10	Genu IV with three setae	**R. (R.) hirtellus Athias-Henriot, Algeria**
–	Genu IV with four setae	**11**
11	Ratios *d1*–*d1/c1*–*c1* = 3.00, *c2*–*c2*/*d1*–*d1* = 3, *d1*–*d1*/*e1*–*e1* = 0.58, *e1*–*e1*/*f1*–*f1* = 0.94–1.00	**R. (R.) neocardinalis Atyeo, The Bahamas**
–	Ratios *d1*–*d1*/*c1*–*c1* = 6.00, *c2*–*c2*/*d1*–*d1* = 1.22, *d1*–*d1*/*e1*–*e1* = 1.38, *e1*–*e1*/*f1*–*f1* = 0.65	**R. (R.) conspicuus (Berlese), Colombia**
12	Dorsal setae comparatively long; setae *c1*, and *d1* crossing bases of *d1* and *e1*, respectively	**R. (R.) khorramabadensis Bagheri, Jafari & Paktinat, Iran**
–	Dorsal setae comparatively short; setae *c1*, and *d1* far behind bases of *d1* and *e1*, respectively	**R. (R.) cardinalis (Ewing), USA**
13	Interscutal membrane with one pair of setae	**Raphignathus (Monoraphignathus) subgen. nov. [14**]
–	Interscutal membrane with more than one pair of setae	**27**
14	Palp femur with two setae; femur I with three setae	**R. (M.) arabicus Gomaa & Hassan, Egypt**
–	Palp femur with three setae; femur I with five or six setae	**15**
15	Femur IV with two or three setae	**16**
–	Femur IV with four setae	**21**
16	Femur IV with two setae	**17**
–	Femur IV with three setae	**19**
17	Genu II with five setae including micro setae	**R. (M.) costatus Chaudhri, Akbar & Rasool, Pakistan**
–	Genu II with six setae including microsetae	**18**
18	Setae *e1* reaching to bases of *h1*; dorsal body setae with spinules along entire length	**R. (M.) zhaoi Hu, Jing & Liang, China**
–	Setae *e1* reaching half distance to bases of *h1* (or distance *e1*–*h1*), dorsal body setae with spinules along entire length	**R. (M.) kuznetzovi Dogan & Ayyildiz, Turkey**
19	Setae *c2* crossing bases of *d1*, Setae *c1* extending to bases of *e1*, setae *e1* extending to bases of *h1*	**R. (M.) ueckermanni Koç & Kara, Turkey**
–	Setae *c2* crossing setae *c1* far behind to the bases of *e1*, setae *e1* far behind to the bases of *h1*	**20**
20	Dorsal body setae ensiform, setae *c1* far behind bases of *d1*, *d1*–*d1* distance almost five times more than *c1*–*c1* distance	**R. (M.) ensipilosus Meyer & Ueckermann, South Africa**
–	Dorsal body setae setiform, setae *c1* far behind bases of *d1*, *d1*–*d1* distance almost equal to *c1*–*c1*	**R. (M.) cometes Atyeo, Bahama-Islands**
21	Small shields absent posterolateral to median prodorsal shield	**22**
–	Small shields present posterolateral to median prodorsal shield	**25**
22	Genital plates/covers with four pairs of setae	**R. (M.) koseiensis Dönel & Doğan, Turkey**
–	Genital plates/covers with three pairs of setae	**23**
23	Femur I and II each with five setae	**R. (M.) solimani Hassan & Gomaa, Egypt**
–	Femur I and II each with six setae	**24**
24	Dorsal setae comparatively long; most setae cross base of next consecutive setae	**R. (M.) kelkitensis Dönel & Doğan, Turkey**
–	Dorsal setae comparatively short; most setae far behind base of next consecutive setae	**R. (M.) fani Doğan & Ayyildiz, Turkey**
25	Dorsal setae comparatively long; most setae reach or cross base of next consecutive setae	**R. (M.) bathursti Meyer & Ryke, South Africa**
–	Dorsal setae comparatively short; most setae far behind base of next consecutive setae	**26**
26	Trochanter III with three setae	**R. (M.) afyonensis Akyol & Koç, Turkey**
–	Trochanter III with two setae	**R. (M.) collegiatus Atyeo, Baker, & Crossley, USA**
27	Interscutal membrane with two pairs of setae	**R. (Diraphignathus) subgen. nov. [28**]
–	Interscutal membrane with three or four pairs of setae	**R. (Triraphignathus) subgen. nov. [60**]
28	Medial prodorsal shield with two pairs of setae	**29**
–	Medial prodorsal shield with three pairs of setae	**30**
29	Setae *c1* present, setae *vi* absents; presence of plates behind the anteromedian plate; femur IV with 2 setae	**R. (D.) evansi Zaher & Gomaa, Egypt**
–	Setae *c1* absent, setae *vi* present; dorsum without а pair of small plates behind anteromedian plate; femur IV with 3 setae	**R. (D.) ehari Zaher & Gomaa, Egypt**
30	Genital plates/covers with four pairs of setae	**31**
–	Genital plates/covers with three pairs of setae	**32**
31	Two small shields posterior to median prodorsal shield absent; endopodal shields absent; setae *f1* behind the anterior margin of opisthosomal shield	**R. (D.) saboorii Ghorbani & Bagheri, Iran**
–	Two small shields posterior to median prodorsal shield present; endopodal shields present; setae *f1* on the anterior margin of opisthosomal shield	**R. (D.) karabagiensis Akyol & Koç, Turkey**
32	Palp femur with two setae	**33**
–	Palp femur with three setae	**44**
33	Two small shields posterior to median prodorsal shield present	**34**
–	Two small shields posterior to median prodorsal shield absent	**38**
34	Endopodal shield present; femur IV with three setae	**35**
–	Endopodal shield absent; femur IV with two setae	**36**
35	Tarsus I with two solenidia	**R. (D.) hecmatanaensis Khanjani & Ueckermann, Iran**
–	Tarsus I with one solenidion	**R. (D.) arcus Akyol, Turkey**
36	Femur IV with two setae; tarsus I with one solenidion	**R. (D.) neohecmatanaensis sp. nov. Alatawi & Kamran, Saudi Arabia**
–	Femur IV with three setae; tarsus I with two solenidia	**37**
37	Lateral prodorsal shield with one pair of pob; tibiae III 5(+1φp) tarsi 18(+1ω+1ω2)	**R. (D.) seraji Pishehvar & Khanjani, Iran**
–	Lateral prodorsal shield without pob; tibiae III 5 tarsi 19(+1ω+1ω2)	**R. (D.) rakhshandehi Pishehvar & Khanjani, Iran**
38	Dorsal setae distally forked or tricarinate	**R. (D.) furcisetosus Meyer & Ueckermann, South Africa**
–	Dorsal setae simple, not distally forked or tricarinate	**39**
39	Femur IV with two setae	**R. (D.) erzincanica Doğan, Turkey**
–	Femur IV with three setae	**40**
40	Opisthosomal shield reduced; interscutal membrane more longer than opisthosomal shield	**41**
–	Opisthosomal shield equally long or longer than interscutal membrane	**43**
41	Dorsal setae stout, serrate and blunt-tipped	**R. (D.) membranus Fan & Yin, China**
–	Dorsal setae simple, distally pointed	**42**
42	Interscutal membrane four times longer than much reduced opisthosomal shield; *f1* on anterior margin of opisthosomal shield	**R. (D.) vahiti Doğan, Turkey**
–	Interscutal membrane slightly longer than opisthosomal shield; *f1* behind anterior margin of opisthosomal shield	**R. (D.) giselae Meyer & Ueckermann, Zimbabwe**
43	Median prodorsal shield anteriorly extending to peritremes and wider anteriorly near setae *sci* as compared to posterior half; setae *f1* on anterior margin of opisthosomal shield	**R. (D.) gracilis (Rack), Germany**
–	Median prodorsal shield anteriorly far behind peritremes and almost equally wide anteriorly near setae *sci* and at posterior half; setae *f1* just behind anterior margin of opisthosomal shield	**R. (D.) bakeri Zaher & Gomaa, Egypt**
44	Small shields posterior to median prodorsal shield absent	**49**
–	Two small shields posterior to median prodorsal shield present	**45**
45	Coxae II with one seta	**R. (D.) atyeoi Meyer & Ueckermann, South Africa**
–	Coxae II with two setae	**46**
46	Femur IV with two setae	**47**
–	Femur IV with three setae	**48**
47	Coxae III and IV with endopodal shields; setae *f1* on posterior margin of interscutal membrane; distance *f1*–*f1* < *d1*–*d1*	**R. (D.) summersi Robaux, USA**
–	Coxae III and IV without endopodal shields; setae f1 far behind posterior margin of interscutal membrane; distance *f1*–*f1* > *d1*–*d1*	**R. (D.) aciculatus Fan, China**
48	Tarsus I–IV 19+ ω, 15+1ω, 13, 12	**R. (D.) africanus Meyer & Ueckermann, South Africa**
–	Tarsus I–IV 21+1 ω, 21+1ω, 15, 14	**R. (D.) hatamii Khanjani & Pishehvar, Iran**
49	Coxa II with one seta	**R. (D.) rarus Kuznetsov, USSR**
–	Coxa II with two setae	**50**
50	Coxae III and IV without endopodal shields	**51**
–	Coxae III and IV with endopodal shields	**53**
51	*c1*–*f1*/*f1*–*f1* = 0.70; *c1*–*f1* < *f1*–*f1*; space between setae *f1*–*f1* twice as wide as between setae *d1*–*d1*	**R. (D.) atomatus Fan & Zhang, New Zealand**
–	*c1*–*f1*/*f1*–*f1* = 1.50–1.87; *c1*–*f1* 1.5–2.0 times more than *f1*–*f1*; distance *f1*–*f1* ≤ *d1*–*d1*	**52**
52	Dorsal setae barbed; setae *c1*, *d1*, and *e1* reach or cross bases of next consecutive setae, distances *d1*–*f1* ≤ *f1*–*f1*; setae *f1* near anterior margin of opisthosomal shield	**R. (D.) satoi Shiba, Malay Peninsula**
–	Dorsal setae simple; setae *c1*, *d1*, and *e1* not reaching base of next consecutive seta; distances *d1*–*f1* 1.31 times more as *f1*–*f1*, *f1* behind anterior margin of opisthosomal shield	**R. (D.) kamiensis Meyer & Ueckermann, South Africa**
53	Femur I and II with five and four setae, respectively	**R. (D.) hexeris Chaudhri, Akbar & Rasool, Pakistan**
–	Femur I and II with six and five setae, respectively	**54**
54	Opisthosomal shield 2–4 times wider than interscutal membrane	**55**
–	Interscutal membrane as wide as or more wider than opisthosomal shield	**56**
55	Setae *c1* far behind posterior margin of prodorsal shield; tibia III with five setae excluding solenidion; opisthosomal shield four times wider than interscutal membrane	**R. (D.) neogracilis Robaux, USA**
–	Setae *c1* on the posterior margin of prodorsal shield; tibia III with four setae excluding solenidion; opisthosomal shield twice as wide as interscutal membrane	**R. (D.) scutatus Kuznetsov, USSR**
56	Femur IV with two setae	**57**
–	Femur IV with three setae	**59**
57	Dorsal shields without striations, tarsus I with 22 setae	**R. (D.) tumidus Kuznetsov, USSR**
–	Dorsal shields with fine, sparse puncta and faint striae; tarsi I with 21 setae	**58**
58	Tarsi III–IV with 14 and 13 setae, respectively; femur II with five setae	**R. (D.) caspicus Doustaresharaf and Kazemi, Colombia**
–	Tarsi III–IV with 15 and 14 setae, respectively; femur II with six setae	**R. (D.) tamaricis Poudineh, Ramroodi & Bagheri, Iran**
59	Setae *f1*–*f1* ≤ *c1*–*c1* and *d1*–*d1*	**R. (D.) giresuniensis Doğan, Turkey**
–	Setae *f1* twice as widely spaced as *c1*–*c1*	**R. (D.) orientalis Fan & Li, China**
60	Medial prodorsal shield with two pairs of setae	**R. (T.) lenis Barillo, Uzbekistan**
–	Medial prodorsal shield with ≥3 pairs of setae	**61**
61	Genital shield with four pairs of setae	**62**
–	Genital shield with three pairs of setae	**63**
62	Two small shields present posterolateral to prodorsal shield; endopodal shields near coxae III and IV absent	**R. (T.) sceptrum Chaudhri, Akbar & Rasool, Pakistan**
–	Small shields absent posterolateral to prodorsal shield; endopodal shields near coxae III and IV present	**R. (T.) quadrigeminus Dönel & Doğan, Turkey**
63	Palp femur with three setae	**R. (T.) aethiopicus (Meyer & Ryke), South Africa**
–	Palp femur with two setae	**64**
64	Femur IV with two setae	**R. (T.) karrooi Meyer & Ueckermann, South Africa**
–	Femur IV with three setae	**65**
65	Femur I with five setae, femur II with four setae	**R. (T.) domesticus Shiba, Japan**
–	Femur I with six setae, femur II with five setae	**66**
66	Two small shields present posterolateral to prodorsal shield	**67**
–	Small shields absent posterolateral to prodorsal shield	**68**
67	Tibiae III with four setae, solenidia absent	**R. (T.) hamooniensis Poudineh, Ramroodi & Bagheri, Iran**
–	Tibiae III with five setae with one solenidion	**R. (T.) larestanensis Bagheri, Akrami & Majidi, Iran**
68	Genu II with four tactile setae; endopodal shields near coxae I–II present	**R. (T.) emirdagiensis Akyol & Koç, Turkey**
–	Genu II with five tactile setae; endopodal shields near coxae I–II absent	**R. (T.) ozkani Doğan, Turkey**

## ﻿Discussion

The taxonomic classification of predatory mites of the genus *Raphignathus* are revised, and for the first time, the genus is divided into four subgenera by considering the morphologically valid, persistent, and prominent characters (Atyeo 1963). The use of subgenera supports the identification of raphignathoid species and will help to avoid designation of new species based on variable characters. *Raphignathusevidus*, *R.hsiufui*, and *R.johnstoni* are considered doubtfully valid. They were described based on size of lateral prodorsal shields and number of setae, but these in these characteristics they resemble immature stages (Fan and Yin 2000). The monotypic genus *Neoraphignathus* was erected based on only the single type specimen with a restricted geographical region and its description is brief. We suspect it might have been described based on the immature stage of a *Raphignathus* species, and we suggest that the type species be revised and more specimens collected to confirm the validity of the species and genus.

## Supplementary Material

XML Treatment for Raphignathidae

XML Treatment for Raphignathus (Raphignathus)

XML Treatment for Raphignathus (Monoraphignathus)

XML Treatment for Raphignathus (Diraphignathus)

XML Treatment for Raphignathus (Triraphignathus)

XML Treatment for Raphignathus (Diraphignathus) neohecmatanaensis

## ﻿Additional information

### Author ORCIDs

Eid Muhammad Khan https://orcid.org/0000-0001-5008-2524

Muhammad Kamran http://orcid.org/0000-0001-6084-203X

Jawwad Hassan Mirza https://orcid.org/0000-0002-1726-4331

Fahad Jaber Alatawi https://orcid.org/0000-0002-6824-2650

### Data availability

All of the data that support the findings of this study are available in the main text.
